# NeoStarling: An Efficient and Scalable Collaborative Blockchain-Enabled Obstacle Mapping Solution for Vehicular Environments

**DOI:** 10.3390/s23177500

**Published:** 2023-08-29

**Authors:** Rubén Juárez, Borja Bordel

**Affiliations:** Department of Informatics Systems, Universidad Politécnica de Madrid, 28031 Madrid, Spain; ruben.juarez@alumnos.upm.es

**Keywords:** VANET, blockchain, authentication, HMAC, InterPlanetary File System, security, data reliability, Intelligent Transportation Systems

## Abstract

The Vehicular Self-Organizing Network (VANET) is a burgeoning research topic within Intelligent Transportation Systems, holding promise in enhancing safety and convenience for drivers. In general, VANETs require large amounts of data to be shared among vehicles within the network. But then two challenges arise. First, data security, privacy, and reliability need to be ensured. Second, data management and security solutions must be very scalable, because current and future transportation systems are very dense. However, existing Vehicle-to-Vehicle solutions fall short of guaranteeing the veracity of crucial traffic and vehicle safety data and identifying and excluding malicious vehicles. The introduction of blockchain technology in VANETs seeks to address these issues. But blockchain-enabled solutions, such as the Starling system, are too computationally heavy to be scalable enough. Our proposed NeoStarling system focuses on proving a scalable and efficient secure and reliable obstacle mapping using blockchain. An opportunistic mutual authentication protocol, based on hash functions, is only triggered when vehicles travel a certain distance. Lightweight cryptography and an optimized message exchange enable an improved scalability. The evaluation results show that our collaborative approach reduces the frequency of authentications and increases system efficiency by 35%. In addition, scalability is improved by 50% compared to previous mechanisms.

## 1. Introduction

In a digitally evolving world, the role of vehicles as communicators within the technological matrix has given rise to Vehicle-to-Everything (V2X) communications. This new communication paradigm, among other innovations, has birthed the Internet of Vehicles, which enables data exchange among vehicles, infrastructure, and the environment [1]. In fact, data exchange among vehicles is the most relevant and promising technology. Therefore, it receives a specific name: Vehicle-to-Vehicle (V2V) communications. Different authors [2] proposed that V2V communication will be essential to improve road safety and traffic efficiency.

V2V communications cannot create fixed networks (as vehicles are mobile) but rather ad hoc networks whose structure evolves dynamically according to the vehicles’ movement. Specifically, Vehicular Ad Hoc Networks (VANETs) and related systems, similar to Mobile Ad Hoc Networks (MANETs), consist of vehicles, Roadside Units (RSUs), and a Trusted Authority (TA) acting as mobile nodes [1] and exchanging data about traffic, each vehicle’s status, etc. These networks, among other advantages, enhance autonomous vehicle decision making by providing environmental data for proactive hazard measures [3]. However, any open communication channel or data exchange has several security risks associated. And V2V networks still face several open challenges, including detecting malicious vehicles, maintaining openness and security, and preventing system-wide failure from a single breakdown point [4]. In particular, techniques to mitigate false data injection in V2V networks in an efficient and scalable manner are required.

In this context, some authors introduced blockchain tools as an innovative solution to improve the visibility of road hazards and data security [5,6]. Among all the previous proposals, the Starling system [7] is the most promising, as it is specifically designed to protect data describing obstacles in the environment. Blockchain forms the core of the Starling system, offering robust authentication and privacy protection. Along with consensus mechanisms such as Proof of Work (PoW), Proof of Stake (PoS), and Proof of Authority (PoA), the system uses smart contracts and the InterPlanetary File System (IPFS) to secure V2V communication processes. As a result, it ensures data integrity and improves obstacle visibility. But, as any other blockchain-enabled tool, Starling faces a critical problem: it is very computationally heavy. But future transportation scenarios will be very dense and dynamic. So, very scalable and efficient alternatives must be investigated.

Therefore, we introduce the NeoStarling system, which is an innovative architecture enhancing the efficiency and scalability of secure blockchain-enabled obstacle-mapping techniques. Our system potentially benefits all network users, improving V2V communication safety and efficiency. NeoStarling incorporates the new and proposed “Secure Vehicular Communication Algorithm” (SVCA), deployed in a permissioned blockchain network, for a robust and efficient scalable secure data exchange. Thanks to HMAC-SHA256 (hash-based message authentication code), NeoStarling redefines V2V communication networks, improving data security, privacy, and network scalability. Although NeoStarling significantly advances V2V communication, some limitations remain. Future developments will explore other complementary technologies such as lightweight nodes, scalable distributed ledger solutions, and potential vehicle data monetization.

The NeoStarling system progresses in three core areas:

- Advanced Cryptography Integration: Bilinear pairing and elliptic curve cryptography (ECC) are combined for robust data security and user authentication. The integration of the Decentralized Secure V2V HMAC-SHA256 Algorithm (DSV-HMAC-SHA256) further enhances security and efficiency.
- Innovative Secure Communication and Re-Authentication Strategy: Our “Secure Vehicular Communication Algorithm” (SVCA) ensures data authenticity and minimizes threats from malicious vehicles, contributing to reliable and secure V2V communication.
- Enhanced Scalability and Efficiency: The implementation of a permissioned blockchain network using PoA increases scalability by approximately 50%, allowing for a larger number of vehicles to share real-time data without network overload.
- Improved Data Reliability and Privacy: Starling guarantees data validity and improves reliability by around 30% using Distributed Ledger Technology (DLT) and blockchain technology. The system protects data from tampering and loss while ensuring equitable access and transparency.

The remainder of this document is organized as follows. Section 2 introduces the state of the art on secure and reliable obstacle detection and mapping. Section 3 details our proposed system, outlining the proposed algorithm and protocol. Section 4 evaluates the proposed technology, examining its performance and scalability, and discussing the obtained experimental results and Section 5 concludes the paper.

## 2. Literature Review

Vehicle-to-Vehicle (V2V) networks, specifically Vehicular Ad Hoc Networks (VANETs), are central to enhancing road safety by improving vehicle visibility, particularly when traditional onboard sensors fall short [8,9]. The crucial role that VANETs play in modern transportation systems is increasingly being recognized, with the ability to facilitate real-time communication between vehicles, improving situational awareness, and thereby enhancing road safety [10]. These networks are fundamentally built upon the exchange of obstacle map data, providing valuable information about potential road hazards, enabling advanced warning systems, and fostering overall safer driving conditions. However, the accuracy, integrity and security of these data are of paramount importance, as any inaccuracies or compromises could lead to incorrect hazard perception and possibly catastrophic consequences [4,11]. Hence, substantial effort is required to maintain these data characteristics while enabling efficient and rapid data exchange [12].

The investigation of obstacle detection for vehicles has a rich history, dating back to the 1980s and 1990s, before the advent of autonomous driving technology [13,14]. Initial techniques focused primarily on obstacle detection to avoid collisions, often neglecting the crucial aspect of data exchange between vehicles. However, technological advances have reshaped this domain. High-resolution cameras and sophisticated sensors such as LIDAR have elevated detection methods, considerably enhancing their reliability and accuracy [15,16]. Consequently, evolved detection methods provide a more comprehensive understanding of the driving environment, contributing significantly to reducing collision incidents [1].

Despite these advances, challenges remain to secure an efficient exchange of obstacle data, which is crucial for the comprehensive functionality of V2V networks. Thus, blockchain technology, known for its security and the decentralization advantages, has seen its application in the V2V communication space. But, still, its potential to enable coordinated obstacle mapping, a significant aspect of VANETs, is often neglected [17,18]. Nevertheless, blockchain has been successfully employed in other VANET subsystems mostly as enabling technology for secure data transmissions or key exchange.

Expanding on exciting and innovative research in blockchain-enabled V2V communication systems, several researchers have presented promising methods and architectures to enhance security and efficiency within VANETs. Shrestha et al. [5] introduced a novel blockchain system that ensures secure message exchange within VANET. The system utilizes blockchain’s immutability and transparency features to validate the authenticity of transmitted messages, thereby improving the trustworthiness of VANET communications. Furthermore, Ma et al. [6] proposed a decentralized key management mechanism that provides robust security in VANET. Leveraging the blockchain’s decentralized nature, the authors built a system that eliminates single points of failure, thereby enhancing the robustness and reliability of key management in VANET. Additionally, Luo et al. [19] present a blockchain-enabled trust-based location privacy protection scheme in VANET. This scheme uses blockchain to create a decentralized, trust-based model that protects user privacy while ensuring secure V2V communication. But none of these solutions is designed to protect obstacle information or ensure efficiency or scalability.

**Figure 1:**
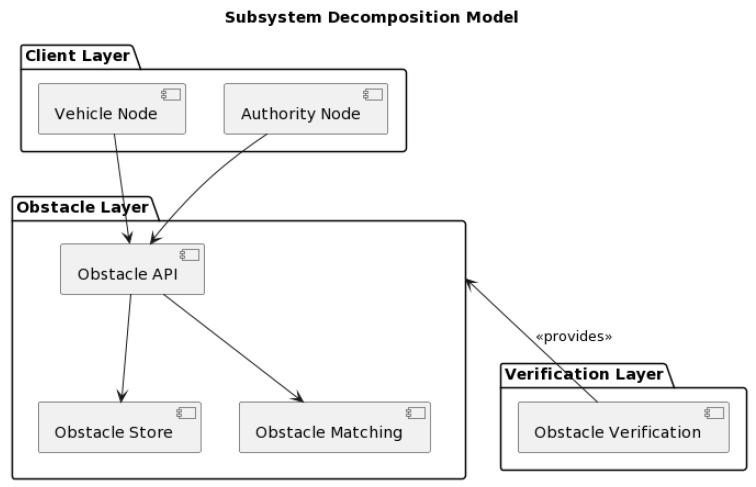
Subsystem decomposition model of the standard Starling system.

Only a very few authors have reported blockchain-enabled secure obstacle mapping solutions in VANETs. The Starling system [7] is probably the most promising and popular. Starling is an innovative solution designed to improve road safety. This system leverages the strengths of blockchain technology, offering secure storage and retrieval of road obstacle data [20]. The system design aims to minimize the traditional problems associated with obstacle data exchange [21], paving the way for safer and more efficient V2V communications. The proposed Starling system is built on an open-layered architecture that encompasses six autonomous subsystems in three hierarchical layers (see Figure 1). This layout provides a structured and efficient network for communication, making the system capable of handling complex V2V communications with ease [22].

**Figure 2:**
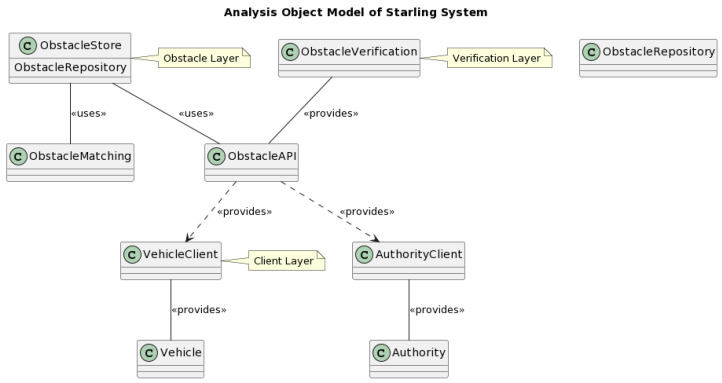
Analysis object model of Starling system.

The Starling system involves three central actors: vehicles, vehicle owners, and enforcement authorities. Each actor has unique roles and requirements within the network, which dictates their unique interaction with the system [23]. Figure 2 represents those interactions. Vehicles communicate with the system via the VehicleClient interface, which is situated within the system’s topmost layer, the Client Layer. This interface allows vehicles to access the Obstacle Repository located in the Obstacle Layer, enabling them to record and retrieve obstacle data [24]. This feature allows for a more dynamic and adaptive navigation system, thereby improving traffic efficiency and safety. An additional utility provided by the VehicleClient interface is the VehicleIdentifier. Enforcement authorities can solicit this identifier during investigations, instilling accountability and encouraging responsible driving behaviors [25]. This accountability measure serves to protect the integrity of the system and enhance the safety it provides.

But Starling and the other prior solutions encountered issues such as high latency in alias generation, inefficient V2V and Vehicle-to-RSU communication due to the limited presence of Roadside Units (RSUs), and increased computational costs from nodes vying to add blocks to the blockchain [26,27]. All these open problems result in a very poor scalability and efficiency, which prevents the implementation of these novel schemes in real transportation applications. These challenges highlight the need for more effective and innovative solutions in this field, and this paper aims to fill this gap.

## 3. Proposed System Design

This section delves into the details of our proposed solution, including the secure algorithms to enhance vehicular communications. Section 3.1 discusses the general overview of the NeoStarling system. Section 3.2 describes the Decentralized Secure V2V HMAC-SHA256 Algorithm employed in the efficient and scalable registration and authentication process. Finally, Section 3.3 presents an optimized and efficient authentication protocol for the NeoStarling system and how it is integrated into the standard Starling architecture.

### 3.1. General Overview of the NeoStarling System

In this section, we discuss the global functionality of the NeoStarling system, which is rooted in the Starling framework. In general, our novel NeoStarling design deploys a custom HMAC-SHA256 algorithm, focusing on enhancing data reliability and data secure communication, directly addressing the prevalent challenges in the V2V communication realm [28]. But, at the same time, this lightweight scheme ensures a significant improvement in the system scalability. Also, this new algorithm allows for an optimized authentication protocol, which increases the global NeoStarling efficiency.

**Figure 3:**
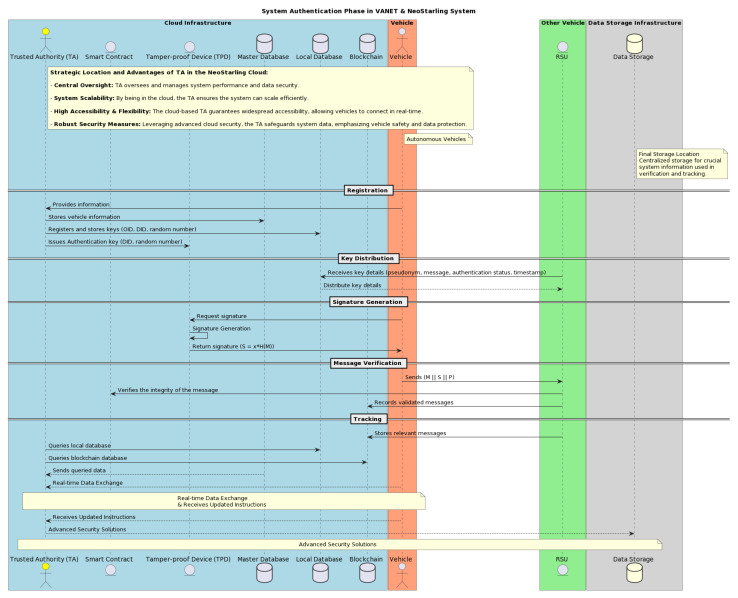
Decomposition model for the proposed NeoStarling system.

Figure 3 represents the decomposition model of the NeoStarling system, detailing the key steps involved in the process and how they interlink to form a secure and efficient V2V communication system.

This illustration also accentuates the strategic location of the Authority Terminal (TA) within the NeoStarling system’s cloud infrastructure together with the tamper-proof device (TPD), master and local databases and the blockchain network (as well as all the deployed SmartContract). The diagram accentuates the benefits of situating the TA (and the remainding components) in the cloud, including system scalability, high accessibility, and robust security measures. This visualization emphasizes the system’s capability to ensure real-time data exchange and seamless communication with autonomous vehicles, underpinning the advancements NeoStarling offers in the realm of V2V communication. As illustrated in the figure, the Authority Terminal (TA) is positioned within the cloud, holding a central role in the NeoStarling system. It ensures optimum system performance and robust data security. By anchoring the TA in the cloud, we capitalize on the myriad benefits of cloud computing.

This includes enhanced scalability, where housing the TA in the cloud allows our system to efficiently scale, smoothly integrating a growing number of vehicles and users without a significant investment in physical infrastructure. It is a testament to the NeoStarling system’s vision of catering to an extensive network of autonomous vehicles. Accessibility and flexibility are also at the forefront with the cloud-based TA guaranteeing widespread accessibility, enabling any vehicle, irrespective of its location, to connect with the TA as long as there is internet connectivity. This pivotal feature ensures real-time data transfer and the receipt of instantaneous directives, which are key elements in the ever-evolving autonomous vehicle network. Lastly, we prioritize security, leveraging the cloud’s advanced security mechanisms to safeguard our system data and amplify vehicle safety. With premier cloud service providers employing advanced security protocols, NeoStarling’s data remain fortified against potential cyber threats, ensuring the NeoStarling system operates both securely and efficiently.

The model divides the NeoStarling operation into five sequential stages, each contributing a unique aspect to the overall performance of the system.

Stage 1—System initialization: This is the stage at which vehicles and Roadside Units (RSUs) are registered with the Trust Authority (TA). The registration process involves the generation of unique identifiers for each vehicle and RSU. This forms the basic structure of the network, establishing the fundamental connections between various nodes within the system [28]. It sets the stage for communication and data exchange, preparing the network for subsequent stages.

Stage 2—Encryption key generation: This stage pertains to the creation of encryption keys necessary for the signing and transmission of secure messages. Utilizing advanced cryptographic techniques, the NeoStarling system generates unique keys for each vehicle and RSU. The keys are used to sign messages and verify their authenticity, ensuring the secure transmission of data across the network.

Stage 3—Message verification: Once the encryption keys have been generated, the NeoStarling system moves to the message verification phase. Here, the authenticity and integrity of the incoming messages are verified [29]. This verification process helps detect and mitigate potential data breaches, thus ensuring the reliability of communicated data. Additionally, it provides a mechanism to trace real identities in case of disputes, enhancing the transparency and security of the system.

Stage 4—Obstacle tracking and data storage: During this phase, the NeoStarling system facilitates the storage and retrieval of obstacle data within a secure repository [30]. The collected data, originating from various vehicles and RSUs, are used to detect hazards in real time, which contributes significantly to road safety. The repository maintains a record of all collected data, which can be accessed and utilized as needed.

Stage 5—Data quality assurance: To ensure data accuracy and quality, the NeoStarling system incorporates checks to prevent the duplication of obstacles within the repository and to detect faulty or manipulated obstacle data. This final step is crucial in maintaining the credibility and reliability of the system, ensuring the provision of accurate and quality information to all users.

**Figure 4:**
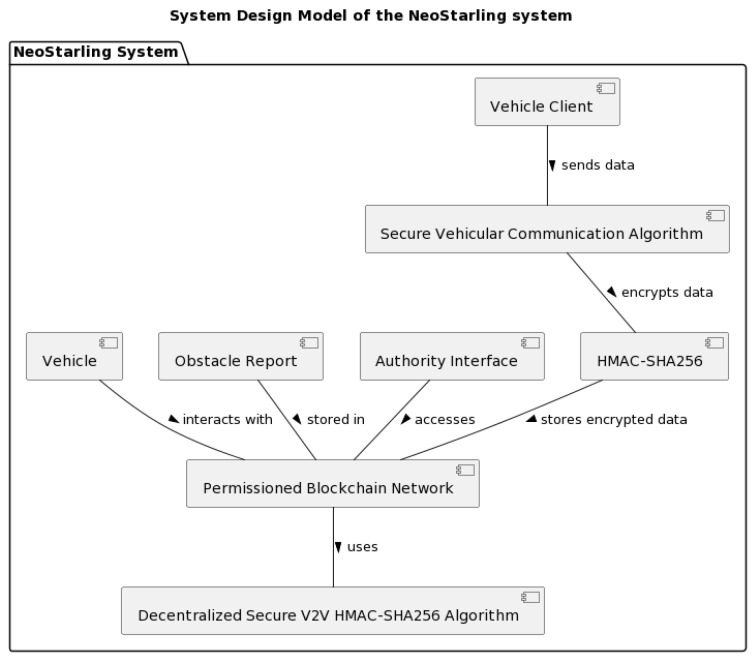
System design model of the NeoStarling system.

**Figure 5:**
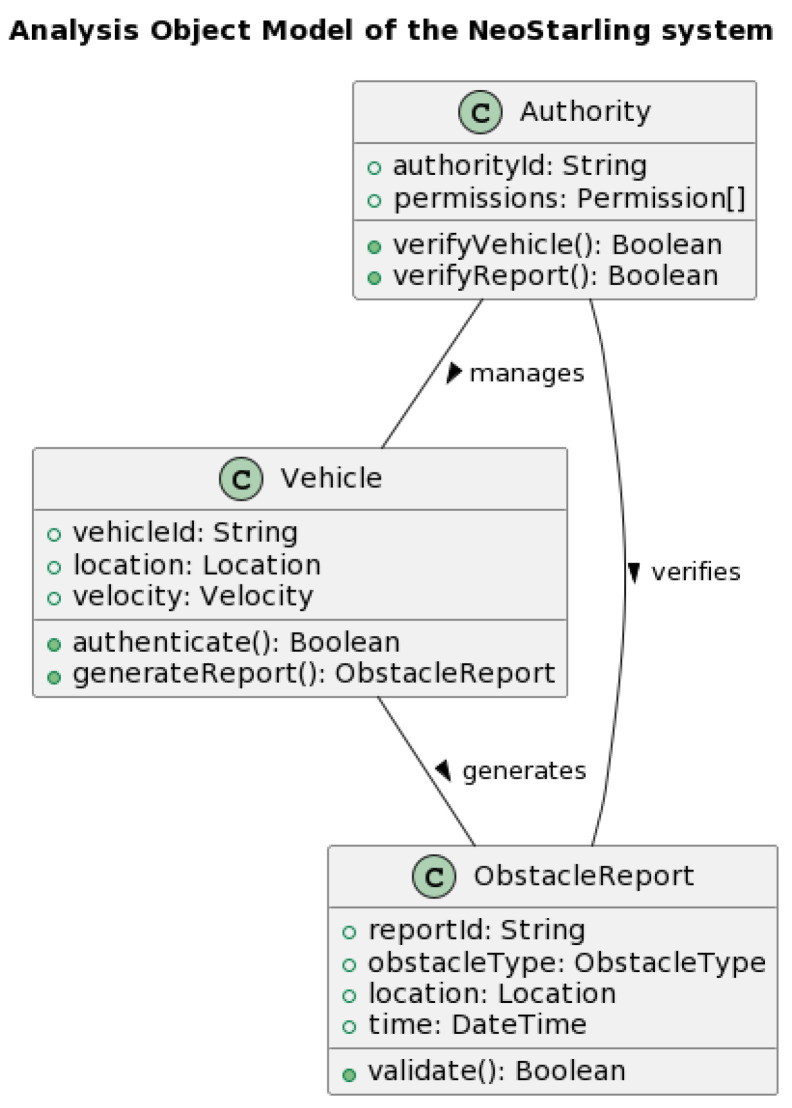
Analysis object model of the NeoStarling system.

Figure 4 and Figure 5 depict the system design model and the analysis object model of the NeoStarling system, respectively. These diagrams demonstrate the progression of the system from its predecessor, the Starling system, and the innovations it brings to the field of V2V communication.

In Figure 4, we see an enhanced version of the Starling system architecture. The crucial additions in the NeoStarling model include a more robust encryption mechanism, the integration of an advanced reauthentication strategy, and the inclusion of an efficient data quality assurance system. These new features amplify the security and efficiency of the system, setting the NeoStarling model apart from its predecessor and the current state of the art [5,6].

Figure 5 presents the Analysis Object Model of the NeoStarling system, outlining its data structure and the interactions among various components. The inclusion of the Secure Vehicular Communication Algorithm (SVCA) and the adoption of HMAC-SHA256 for data integrity verification are key differentiating features. These additions enable the NeoStarling system to ensure data authenticity while minimizing threats from malicious vehicles, contributing to reliable and secure V2V communication [19].

These improvements not only enhance the system’s capabilities but also address some of the key challenges faced by contemporary V2V communication systems. The NeoStarling system offers an advanced solution to the growing demand for secure, efficient, and scalable vehicular communication systems.

### 3.2. Decentralized Secure V2V HMAC-SHA256 Algorithm

One of the key innovations of the NeoStarling system, allowing an improved scalability and a higher effciency, is the inclusion of the Secure Vehicular Communication Algorithm (SVCA) and the adoption of HMAC-SHA256 for data integrity. The combination of these two technologies is named “DSV-HMAC-SHA256”.

The proposed DSV-HMAC-SHA256 algorithm is a cryptographic communication protocol specifically tailored to the demands of Vehicle-to-Vehicle (V2V) communications. This algorithm streamlines the registration and authentication of vehicles and Roadside Units (RSUs), the generation of temporary keys when entering the RSU range, the creation of short-term anonymous signatures, message verification, and even dispute resolution [31]. By incorporating DSV-HMAC-SHA256 into our NeoStarling system, we can provide secure, efficient, and reliable communications [32] between vehicles, improving obstacle detection and reporting capabilities. Ultimately, this algorithm improves the overall safety and functionality of our NeoStarling system, bolstering the trust of our users and stakeholders in the integrity, reliability and security of the system [33].

In the context of a vehicular network environment, several key considerations must be made when selecting a hash algorithm. These include security, computational efficiency, and data transmission speed. The choice to utilize SHA256 in NeoStarling was largely based on striking a balance between security and computational efficiency. In terms of security, although SHA256 provides a smaller hash size compared to SHA512, it still delivers a suitable level of security for many applications. While brute force attacks are theoretically more feasible against SHA256 than against SHA512 due to its smaller hash size, the reality is that brute force attacks against SHA256 remain impractical with current technology. Regarding the computational efficiency, the extended hash size in SHA512 implies more intensive computations, potentially leading to greater computational resource demands and extended processing times. In general, SHA256 is less computationally intensive than SHA512. This is particularly important in a vehicular network environment where computing resources may be limited and efficiency is critical. More efficient hash algorithms allow for faster processing, which can be crucial for real-time operations. Regarding data transmission, by using SHA256, the size of the hashes (and therefore, the size of the transmitted data) is smaller than with SHA512. This can contribute to faster data transmission and less bandwidth usage, which can be beneficial in a vehicular network environment. In conclusion, while SHA512 may offer theoretically greater security, NeoStarling uses SHA256 because it still provides a formidable security level, has lower demand on computational resources and contributes to more efficient data transmission.

**Figure 6:**
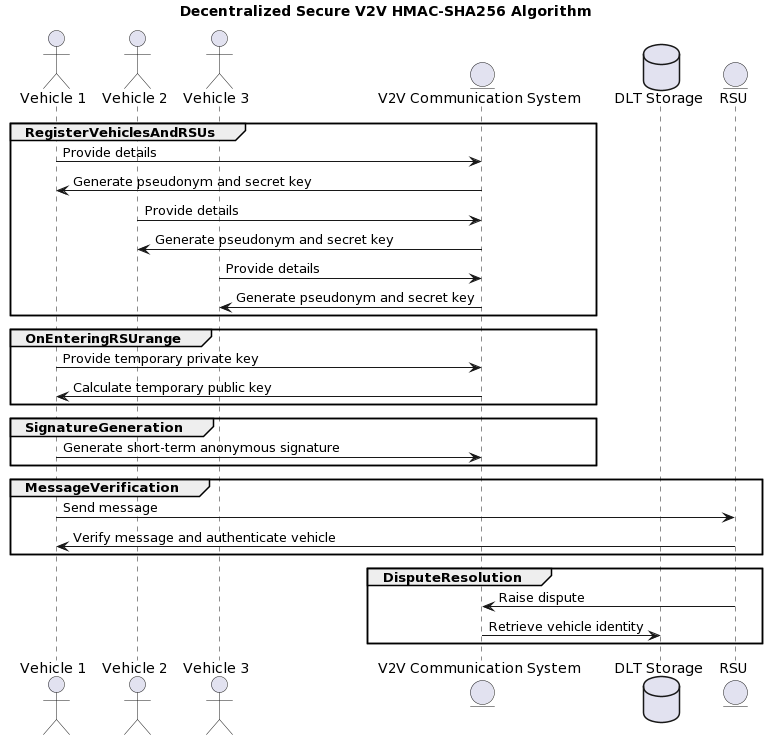
Secure decentralized V2V HMAC-SHA256 algorithm.

Figure 6 illustrates the secure decentralized V2V HMAC-SHA256 algorithm (DSV-HMAC-SHA256) in a message exchange chart.

It is important to highlight that the Roadside Unit (RSU) generates distinct ledgers for each vehicle within the network. This approach offers manifold benefits, particularly with respect to enhancing data security, preserving privacy, and ensuring efficient data management. Each vehicle is associated with a uniquely tailored ledger. This customization fortifies the data isolation for each vehicle and provides robust security, thereby minimizing potential exposure to unauthorized entities. This approach plays a critical role in enhancing the privacy of vehicular data and mitigating potential security breaches. From a data management perspective, having a dedicated ledger for each vehicle significantly streamlines the process of pinpointing and retrieving specific information as required. This means that if data pertaining to a specific vehicle are needed for analysis, the system can directly access the associated ledger without the necessity of sifting through extensive data from a multitude of vehicles. It is worth noting that while every vehicle possesses its own private ledger, synchronization and consistency across all these ledgers are maintained via blockchain consensus mechanisms. This feature guarantees the preservation of data integrity and ensures consistency across the entire vehicular network, thereby providing a unified and precise snapshot of the network’s status at any given point. The RSU’s creation of private ledgers for each vehicle is a cornerstone of our system architecture, bolstering security, enhancing privacy, and ensuring efficient data management within the NeoStarling vehicular network.

As can be seen, five different interconnected procedures make up the global DSV-HMAC-SHA256 protocol: RegisterVehiclesAndRSUs, OnEnteringRSUrange, SignatureGeneration, MessageVerification and DisputeResolution. From the cryptographic point of view, in our NeoStarling system, we implement various methods for data authentication and security. The next subsections describe all these methods and procedures in detail.

#### 3.2.1. Register Vehicles and RSUs

In this procedure, every vehicle or Roadside Unit (RSU) in the network is registered with a unique pseudonym and a secret key. This process involves collecting the necessary details and generating unique pseudonyms and secret keys for each entity [34]. Algorithm 1 describes this procedure in pseudocode. The Intelligent Roadside Units (RSUs) generate their own private ledgers, which store details received from neighboring RSUs, like the vehicle’s pseudonym, its message, authentication status, and timestamp. RSUs generate authentication parameters through bilinear pairing for elliptic curves. This implies that the vehicle selects a random number as a short-term private key and computes a corresponding short-term public key on the elliptic curve. The RSU verifies whether the vehicle is authenticated or not. If the vehicle is not authenticated, the RSU asks for authentication parameters (see Section 3.3); otherwise, it passes the “successful authentication” status.

Our proposed vehicular network system, NeoStarling, fundamentally relies on the generation of public and private keys by the Roadside Unit (RSU). The RSU’s key generation serves as a cornerstone to ensure data integrity and to strengthen the system’s security structure. We have consciously chosen a 256-bit key, as even though longer keys could potentially provide greater security, they would also intensify the computational and transmission demand. Given the dynamic and critical nature of vehicular networks, efficiency is paramount. After thorough analysis, we have determined that 256-bit keys offer adequate security without compromising this essential efficiency. Regarding the role played by the RSU, it is responsible for generating private records for each vehicle, focusing on its unique identity and driving behavior. Each vehicle within the NeoStarling network is assigned a unique identifier, which facilitates the tracking and maintenance of individual records, significantly enhancing the privacy and security of the data. Moreover, the highlighted aspects of each vehicle’s driving behavior, such as speed, direction, and other pertinent characteristics, are meticulously recorded in the private ledger, enabling thorough and precise tracking. However, although these criteria have been set with the specific needs of the NeoStarling system in mind, they have been designed with the necessary flexibility to adapt to changing objectives and requirements, ensuring the system remains relevant across a wide variety of situations and conditions.

**Figure 7:**
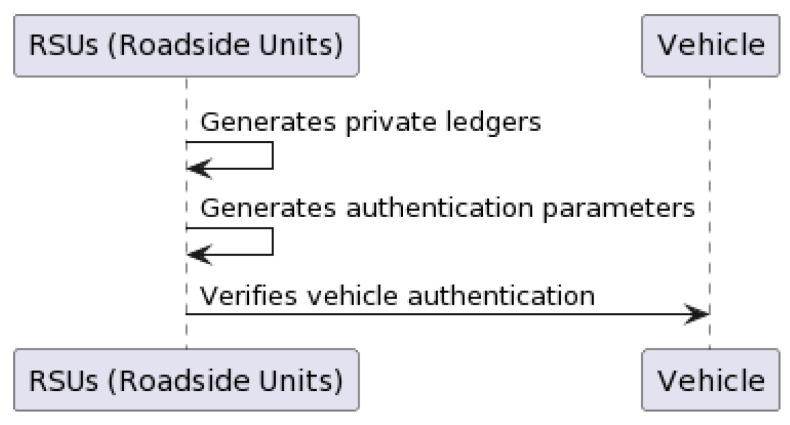
Key generation.

Figure 7 and Algorithm 1 show this procedure as a flow chart and algorithm, respectively.
**Algorithm 1** Register Vehicles And RSUs**Input:** 
Details of vehicle and RSU**Output:** 
Unique pseudonyms and secret keys for each user1:**for each** Vehicle/RSU **do**2:    Collect necessary details3:    Generate unique pseudonyms and secret keys4:**end for**

#### 3.2.2. On Entering RSU Range

When a vehicle enters the range of an RSU, it generates a temporary private key and calculates the corresponding public key. These keys secure communication with the RSU. This key generation process enhances the system’s security by ensuring that the same key is not used in successive sessions, thus minimizing the risk of key compromise [35]. Algorithm 2 describes this procedure in pseudocode.
**Algorithm 2** On Entering RSU Range**Input:** 
Details of vehicle**Output:** 
Temporary private and public key1:**for each** Vehicle **do**2:    Generate a temporary private key3:    Calculate a temporary public key from the private key4:**end for**

In both Algorithms 1 and 2, we integrate elliptic curve cryptography (ECC) algorithms to engender unique and unpredictable keys with each iteration. The strength of these algorithms lies in their reliance on complex mathematical problems, which, as of the present time, have no known efficient solutions. This complexity imbues our key generation process with a high degree of security. Our NeoStarling system harnesses the power of asymmetric cryptography, which employs a public–private key pair. But NeoStarling also adopted the use of ephemeral, or temporary, keys. Characterized by their limited lifespan and frequent renewal, these keys limit the potential damage of any single key being compromised, mirroring protocols such as Kerberos, which are known for utilizing time-limited tickets for authentication. Key transmission between vehicles and Roadside Service Units (RSUs) is conducted through encrypted communication channels fortified by the TLS (Transport Layer Security) standard. This measure provides protection against key interception and “man-in-the-middle” type attacks. To safeguard stored keys, we employ secure isolation techniques. Hardware Security Modules (HSMs) offer robust protection against both system and hardware-level attacks. Finally, our rigorous access control measures encompass multi-factor authentication and role-based authorizations, aligning with best-practice information security principles, such as the least privilege principle. This ensures that key access is limited to authorized entities only. To maintain accountability and traceability, we conduct routine audits of all interactions within the key system.

#### 3.2.3. Signature Generation

**Figure 8:**
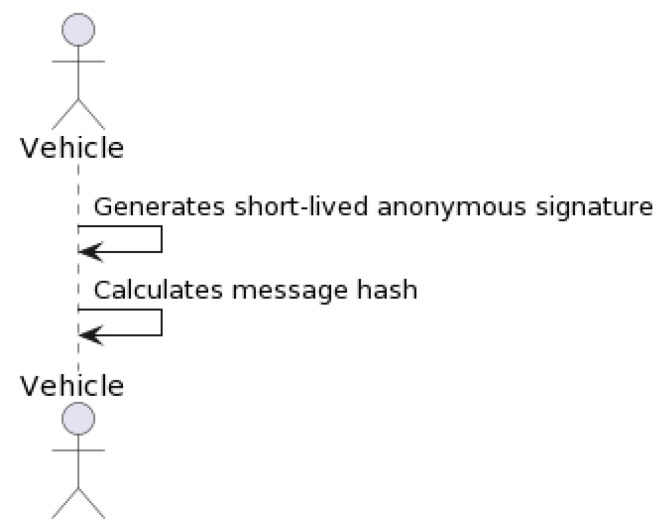
Signature Generation.

To preserve the integrity of the message, in this procedure, each vehicle RRHH generates a short-term anonymous signature using the temporary private key generated in the previous step. This signature authenticates messages sent to the RSU, reducing the likelihood of unauthorized data access and manipulation [36]. Algorithm 3 describes this procedure in pseudocode, while Figure 8 represents the associated flow chart. The vehicle calculates the message’s hash using the HMAC-SHA256 algorithm and maps it to a point on an elliptic curve. The message signature is calculated as shown in Section 3.2.7.
**Algorithm 3** Signature Generation**Input:** 
Details of vehicle, temporary private key**Output:** 
Short-term anonymous signature1:**for each** vehicle **do**2:    Generate a short-term anonymous signature using the temporary key3:**end for**

#### 3.2.4. Message Verification

Upon receiving a message from a vehicle, an RSU attempts to authenticate the vehicle and verify the integrity of the message. If the verification is successful, the RSU sends a receipt back to the vehicle to acknowledge successful transmission [32]. Algorithm 4 describes this procedure in pseudocode.

In summary, the vehicle sends the message, the signature and the public key to the RSU in the form of a concatenated message (Equation (1)). In this context, *M* denotes the message sent by the vehicle, *S* is the signature created by the vehicle’s private key, and *P* represents the vehicle’s public key. The vehicle sends these three pieces of information—*M*, *S*, and *P*—together in a concatenated form to the RSU. This can be represented as follows:

(1) (M||S||P)

Here, the “||” symbol represents concatenation, combining the message, the signature, and the public key into a single string for transmission.

The message is prioritized according to the message type *M*. Different types of *M* could include emergency messages, safety messages, traffic information messages, control messages, and service messages. The system gives the highest priority to emergency messages, which is followed by safety messages and traffic information messages. Control messages receive lower priority, while service messages are typically given the lowest priority.

**Figure 9:**
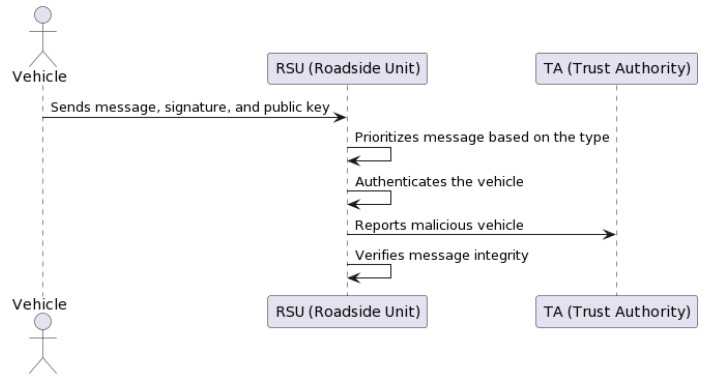
Message verification.

The RSU authenticates the vehicle if a lightweight logic proposition is true (Equation (2)). Otherwise, it is considered a malicious vehicle, and the RSU reports to the TA. The integrity of the message is also verified using the HMAC-SHA256 algorithm (see Section 3.2.8). Figure 9 shows the message verification procedure in a flow chart.

(2) e(G,S)=e(P,H(M))

**Algorithm 4** Message Verification
**Input:** 
Vehicle’s message, Details of RSU**Output:** 
Receipt of successful transmission1:**for each** RSU, Upon receiving a vehicle’s message **do**2:    Authenticate the vehicle3:    Verify the message4:    **if** verification is successful **then**5:        Send a receipt back to the vehicle6:    **end if**7:
**end for**



In Equation (2), *G* is a generator point on an elliptic curve, *S* is the signature sent by the vehicle, and *P* is the public key of the vehicle. The hash of the message *M* is denoted by H(M). The function e() denotes a pairing function on the elliptic curve. The equality e(G,S)=e(P,H(M)) verifies that the signature *S* was generated by the owner of the public key *P*, confirming the authenticity of the vehicle and the integrity of the message.

#### 3.2.5. Dispute Resolution

**Figure 10:**
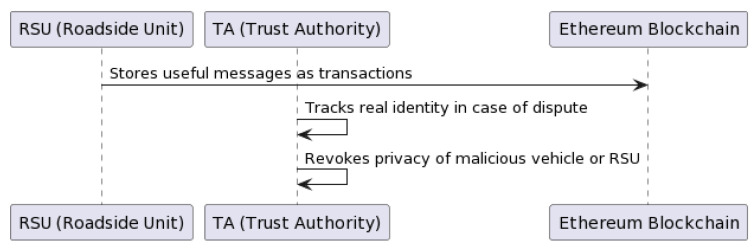
Dispute resolution.

In the event of a dispute, the procedure involves revealing the true identity of the involved vehicle or RSU. This is achieved by looking up the entity’s true identity in the database of the Trust Authority (TA) [37]. Algorithm 5 describes this procedure in pseudocode. RSUs store useful messages on the Ethereum blockchain using the Proof-of-Authority (PoA) consensus algorithm in the form of transactions. In case of dispute, the TA tracks the true identity by looking into its local database and the blockchain database. After tracking the real identity, the TA revokes the privacy of the malicious vehicle or RSU to prevent further harm. Figure 10 shows the dispute resolution procedure in a flow chart.
**Algorithm 5** Dispute Resolution**Input:** 
Details of dispute**Output:** 
Identity of the concerned vehicle or RSU1:**for each** Dispute **do**2:    Reveal the true identity of the concerned vehicle or RSU from the TA’s database3:**end for**

The procedures above aim to increase the system’s reliability and security while addressing specific challenges associated with decentralized systems such as scalability and privacy. The DSV-HMAC-SHA256 algorithm provides secure, authenticated communication between vehicles and RSUs while maintaining the vehicle identity as private. Temporary keys and anonymous signatures safeguard the identities of vehicles, while the option to reveal the true identity in disputes ensures accountability.

#### 3.2.6. Bilinear Pairing

Bilinear pairing is employed in our system to authenticate and correlate the data of detected obstacles, reducing data redundancy and avoiding duplicate entries. Furthermore, an elliptic curve cryptosystem is utilized to maintain the privacy and security of data through a public and private key pair. The pairing function operates between elements of two cyclic groups and outputs a value in a third group. Our system combines this technique with HMAC-SHA256 and IPFS (integrated in the standard Starling system) to secure obstacle detection and reporting.

#### 3.2.7. Elliptic Curve Cryptography (ECC)

ECC is leveraged for generating public keys in our NeoStarling system. ECC, founded on elliptic curve theory, yields cryptographic keys that are efficient, quick, and compact. The cryptosystem works on the principle of ‘easy to compute, hard to reverse’, and it is used to generate a private key (*x*) and a point on the elliptic curve (*G*) whose multiplication results in the public key, as shown in Equation (3):

(3) P=x·G

The key pair produced by ECC forms a critical part of the data authentication and encryption process, ensuring the secure sharing and storing of obstacle data.

#### 3.2.8. HMAC-SHA256 Algorithm

The HMAC-SHA256 algorithm is used to authenticate obstacle information messages, ensuring the reliability of the data. HMAC-SHA256 is a type of keyed-hash algorithm created from the SHA256 hash function. In NeoStarling, it is used for vehicle authentication and message authentication. In the HMAC method, the message is combined with the secret key and processed with the SHA256 hash function, the resulting output is combined again with the secret key, and then the SHA256 hash function is applied again to obtain a 256-bit output hash length. In NeoStarling, to generate an HMAC (Hash-based Message Authentication Code), we use the following mathematical scheme (Equation (4)):

(4) $$\mathrm{HMAC}\left(k,m\right)=H\left(\left(k⊕\mathrm{opad}\right)||H\left((k⊕\mathrm{ipad})||m\right)\right)$$

where:

- *H* is a cryptographic hash function, in this case, SHA256.
- *k* is the secret key used for message authentication.
- *m* is the message.
- opad is the “outer padding” (5c5c5c...5c in hexadecimal).
- ipad is the “inner padding” (363636...36 in hexadecimal).
- ⊕ is the XOR operator.
- || represents concatenation.

**Figure 11:**
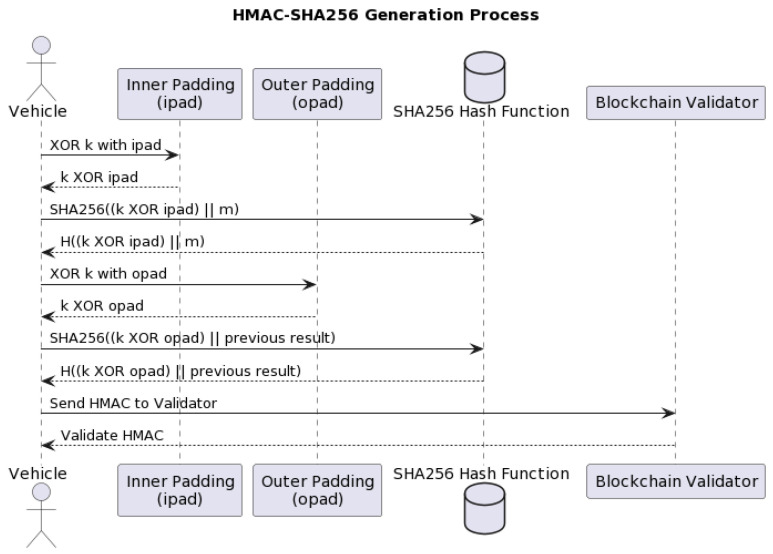
HMAC-SHA256 lifecycle.

Figure 11 shows the HMAC-SHA256 lifecycle. In this figure, the “Vehicle” actor represents the vehicle sender in the system. “Inner Padding (ipad)” and “Outer Padding (opad)” represent the inner and outer padding constants. The “SHA256 Hash Function” represents the cryptographic hash function used. And the “Blockchain Validator” represents the validator in the blockchain system, which validates the HMAC.

The interactions among the components of the NeoStarling system reinforce data security and authentication. The “Vehicle” generates an HMAC using the secret key and the message, employing the HMAC-SHA256 algorithm. This HMAC is verified by the “Blockchain Validator”, ensuring its authenticity before allowing any other entity access to the data. Therefore, this process guarantees the integrity and authentication of the messages within the vehicular communication system.

### 3.3. An Optimized and Efficient Blockchain-Enabled Authentication Protocol

In a Vehicle Ad-hoc Network (VANET), vehicular communication plays a crucial role in mitigating traffic accidents and congestion. Nevertheless, the importance of maintaining the integrity and authenticity of messages escalates, especially for security and privacy purposes. Notably, in Vehicle-to-Vehicle (V2V) communication, while message confidentiality may not be a priority as vehicles only transmit messages after authentication from Roadside Units (RSUs), it becomes essential to enable quick and efficient vehicle authentication. This reduces idle time when vehicles need to communicate with each other and increases the efficiency.

Many proposed systems have centered on diminishing authentication time per RSU, concurrently preserving vehicle security and privacy. It is important to note that vehicles should authenticate each time to prevent malicious vehicle penetration into the system. Prolonged vehicle authentication can create system issues. Thus, our proposed system somewhat curtails the frequency of authentications, reducing authentication delay and facilitating communication with other vehicles.

For acquiring vehicle message history, blockchain proof-of-work technology is considered. However, this demands a high computational cost for each RSU since they must compete to add blocks to the blockchain. As such, our system uses blockchain proof-of-authority technology to cut computational costs. Moreover, to optimize storage, only crucial vehicle messages are stored on the blockchain. This includes emergency messages from ambulances, fire trucks, and other vehicles transmitting information about traffic accidents and congestion. Vehicles are then authenticated and prioritized at RSUs based on the message type.

However, a dense network can pose challenges. As the number of vehicles increases, RSUs experience a higher load, slowing the system. Our scheme alleviates this by reducing the number of authentications, rendering the system relatively faster.

**Figure 12:**
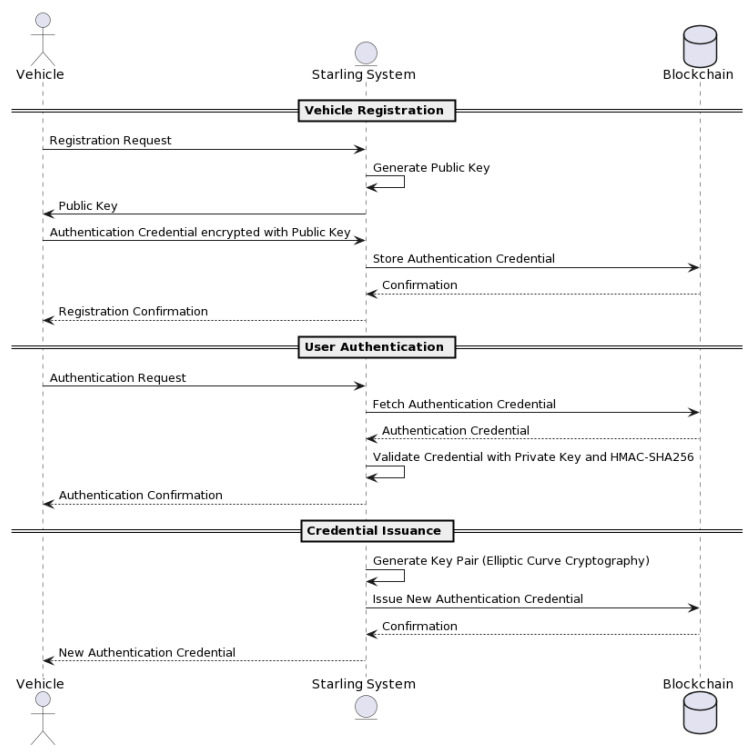
The authentication system in the NeoStarling system.

The DSV-HMAC-SHA256 authentication can be decomposed into the vehicle registration, user authentication, and credential issuance methods (see Figure 12).

The authentication process begins when a vehicle is first registered with the NeoStarling system (see Figure 12). In the initial stage of the registration process, the Starling system generates a public key that is sent to the vehicle. Following this, the vehicle uses this public key to encrypt its authentication credential, which is then sent to the system during the “offer-credential” process.

This procedure provides the vehicle with a secure way to communicate its credentials to the Starling system. The encrypted authentication credential is stored in the blockchain for future reference, thus adding a level of security to the vehicle registration process. Once the registration is confirmed, the vehicle is officially registered and can begin the user authentication process.

**Figure 13:**
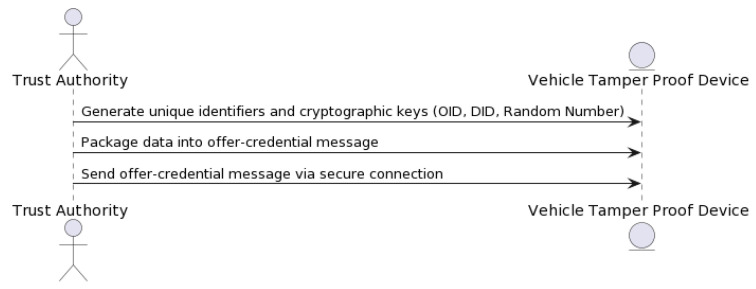
Offering the credential.

Please refer to Figure 13 for a detailed illustration of the “offer-credential” process. This figure depicts the sequence of actions that occur when a vehicle owner provides an authentication credential encrypted with NeoStarling’s public key during the registration request. This approach ensures the security and integrity of the registration process, making it more resilient against potential security threats.

Before registering a vehicle, the “offer-credential” process (see Figure 13) would ensure that the vehicle owner provides an authentication credential encrypted with NeoStarling’s public key, which would be sent alongside the registration request. This process adds an additional layer of security to vehicle registration.

**Figure 14:**
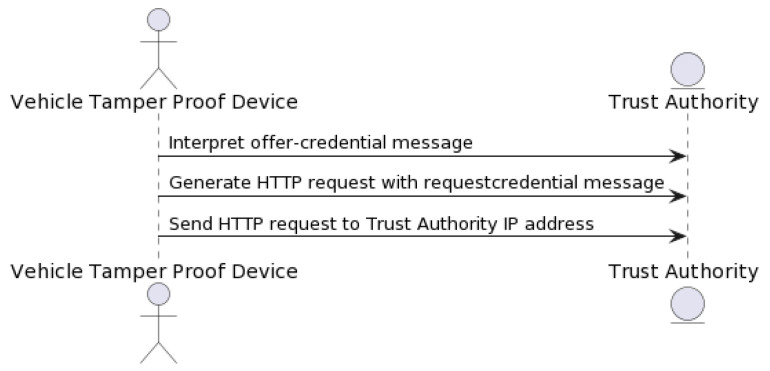
Request credential.

For user authentication, the “request-credential” process can be employed, where users provide an authentication credential. NeoStarling would then decrypt and validate the credential using its private key and the HMAC-SHA256 algorithm. Only authenticated users with valid credentials would be granted access to the system’s services, enhancing security measures. Upon receiving the offer-credential message, the TPD and the onboard agent process the information. The user is informed via an interface on the vehicle. If the user accepts the offer, the agent then formulates an HTTP request containing a “requestcredential” message (see Figure 14). This request is sent back to the Trust Authority, again using the service field in the message to determine the correct IP address for communication.

**Figure 15:**
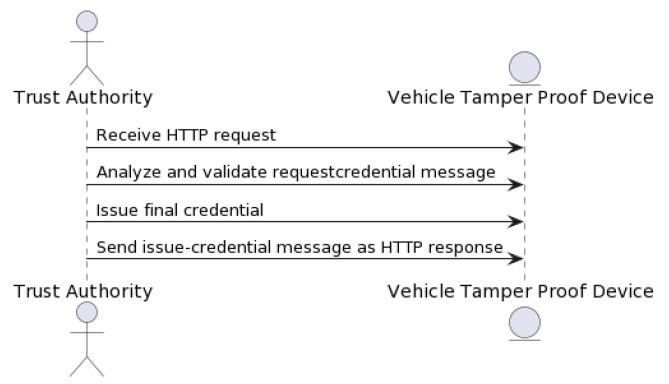
Issue credentials.

In the “issue-credential” process, authorities can issue new authentication credentials to users. This process involves generating a key pair using elliptic curve cryptography, with the private key assigned to the issued credential. The Trust Authority receives the HTTP request (see Figure 15), thoroughly analyzes the requestcredential message, and confirms its authenticity using the elliptic curve cryptography and HMAC-SHA256 algorithms, as described earlier. Once validated, the Trust Authority issues the final credential in accordance with the predefined schema. This credential is then packed into an issue-credential message and returned as a response to the vehicle’s HTTP request. The credential, now stored in the vehicle’s TPD, will be used for future authentications as the vehicle interacts with the decentralized data storage and the blockchain-based network.

The next subsections describe all the methods making up the proposed blockchain-based authentication protocol with details.

#### 3.3.1. Vehicle Registration Process

**Figure 16:**
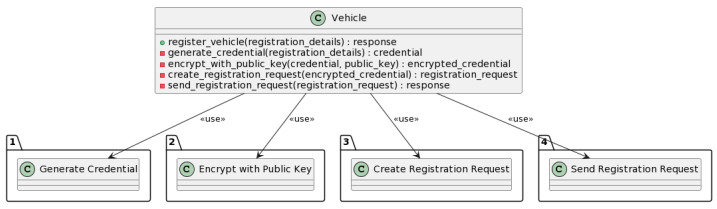
Vehicle registration.

The vehicle registration process involves registering a vehicle in the system with enhanced security measures. Figure 16 describes all the submethods included in the registration phase.

The register_vehicle method is used to register a vehicle with the NeoStarling system. The method takes the vehicle registration details as input and generates a credential for the vehicle. The credential is a unique identifier that is used to identify the vehicle in the NeoStarling system. The credential is encrypted using the public key of the NeoStarling system. This ensures that the credential can only be decrypted by the NeoStarling system.

**Figure 17:**
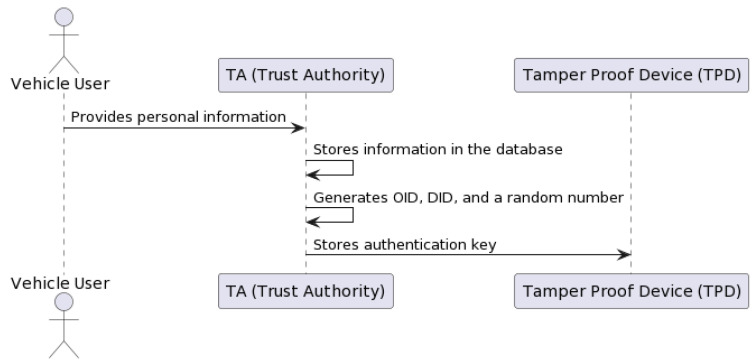
Vehicle registration process.

More specifically, as shown in Figure 17, users go to the Trust Authority (TA) and provide personal information, such as phone number, driver’s license number, vehicle number, etc. This information is stored in the TA’s master database. Using this information, the TA generates unique keys required for each vehicle’s user through the key generation process, including the generation of the original user identity (OID), the pseudonymous user identity (DID), and a random number stored in the local database. The mapping of original identities to pseudonymous identities is completed solely in the TA. Following this, the authentication key, consisting of a pseudonymous identity and a random number, is stored in the vehicle’s Tamper-Proof Device (TPD). The pseudoanonymous identity protects vehicle privacy. This information is then packed into an offer-credential message and sent to the vehicle’s Tamper-Proof Device (TPD) via a secure connection. The connection is set up based on the service field in the message, which specifies the IP address for direct communication with the TPD.

Then, a registration request is created with the encrypted credential (Figure 17). The registration request is sent to the NeoStarling system. The NeoStarling system validates the registration request and returns a response. The response from the system is returned as the output.

In the vehicle registration process, the DSV-HMAC-SHA256 algorithm is integrated into the step of generating the encrypted credential. After generating the credential using the generate_credential function, the DSV-HMAC-SHA256 algorithm would be applied to ensure the integrity of the credential before encrypting it with NeoStarling’s public key.

#### 3.3.2. User Authentication Process

**Figure 18:**
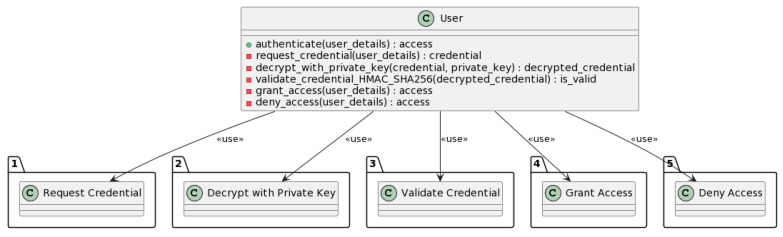
User authentication process.

The user authentication process ensures that only authenticated users can access the system’s services. Figure 18 describes all the submethods included in the authentication process.

The authenticate method is used to authenticate a user with the NeoStarling system. The method takes user details as input and requests a user credential. The credential is a unique identifier that is used to identify the user in the NeoStarling system. The credential is encrypted using NeoStarling’s private key. This ensures that the credential can only be decrypted by NeoStarling. The credential is then decrypted using NeoStarling’s private key. The validity of the decrypted credentials is checked using the HMAC-SHA256 algorithm. If the credential is valid, access to the system’s services is granted. If the credential is invalid, access is denied. The access status is returned as the output.

In the user authentication process, the DSV-HMAC-SHA256 algorithm takes the same role as in the vehicle registration process. After decrypting the credential using NeoStarling’s private key in the decrypt_with_private_key function, the DSV-HMAC-SHA256 algorithm is used to verify the integrity of the credential. This step is performed using the validate_credential_HMAC_SHA256 function.

#### 3.3.3. Credential Issuance Process

**Figure 19:**
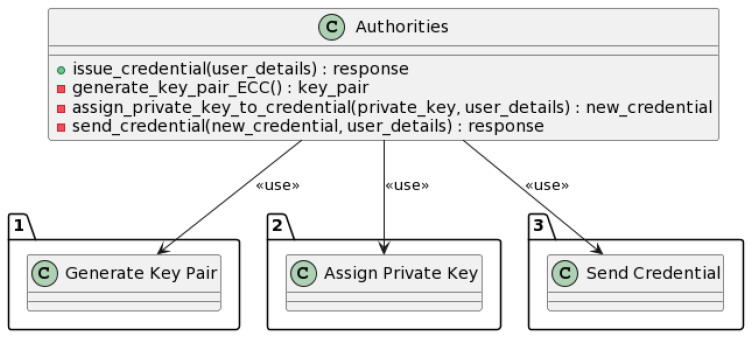
Credential issuance process.

The credential issuance process allows authorities to issue new authentication credentials to system users. Figure 19 describes all the submethods included in the credential issuance process.

The issue_credential method is used to issue a credential to a user.

The method takes the user details as input and generates a key pair using ECC.

The private key from the key pair is assigned to the new credential. The new credential is then encrypted using the user’s public key. This ensures that the credential can only be decrypted by the user.

The new credential is then sent to the user. The user can then use the credential to access the system’s services. The response of the user is returned as the output.

After a successful authentication, our solution leverages OrbitDB and Ethereum for decentralized data storage implementation and for establishing a blockchain network, respectively.

OrbitDB, a distributed database built on IPFS, caters to our database requirements. Its distinctive feature is the conflict-free replicated data structure (CRDT) that ensures node consistency in our distributed environment.

**Figure 20:**
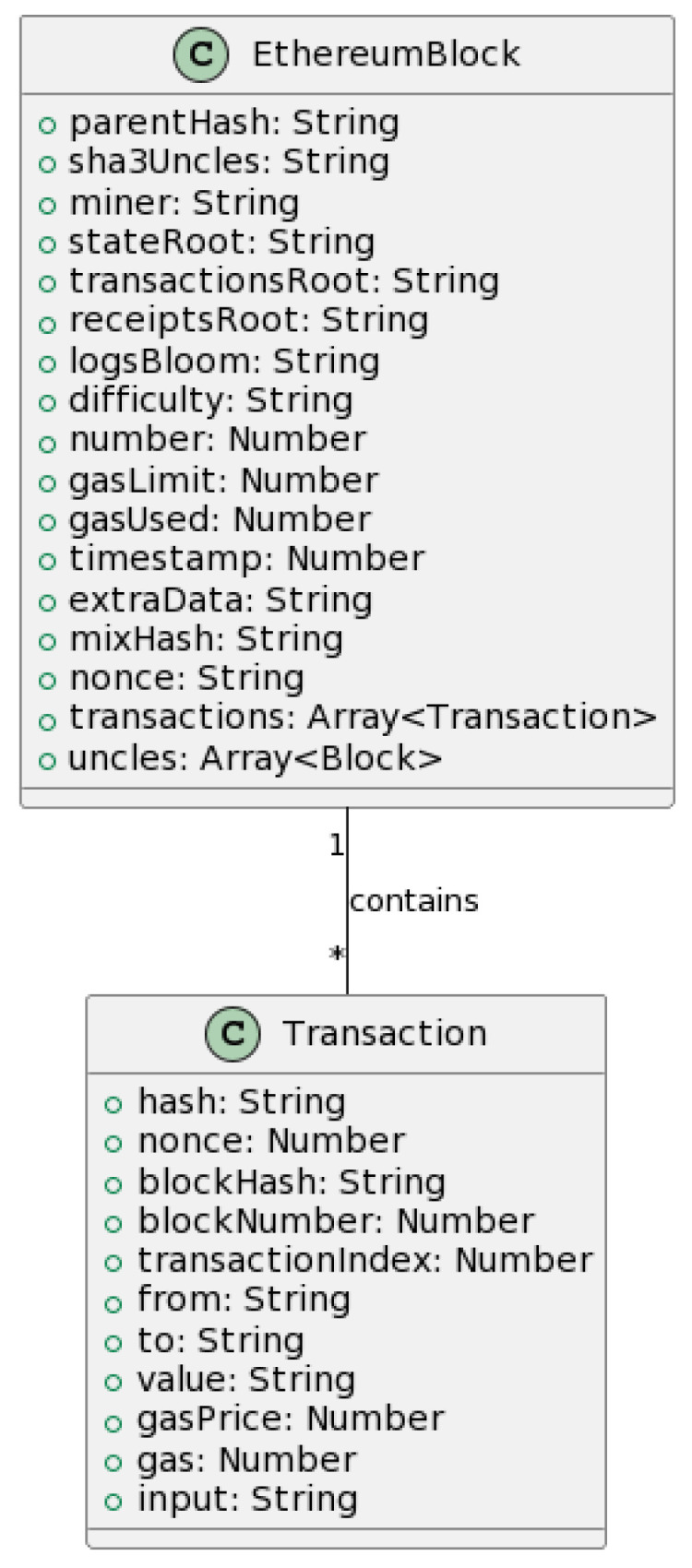
Ethereum block structure in NeoStarling.

The blockchain network is based on Ethereum, which is a versatile and permissionless blockchain capable of executing smart contracts. Ethereum redefines the conventional block structure, introducing unique elements such as Uncles, New Hash Trees, and the concept of Gas (see Figure 20).

To access Ethereum’s test network, NeoStarling opts for Geth client, granting client access through the universally accepted JSON RPC API.

#### 3.3.4. Integration with Standard Starling System

A smart contract is created on the Ethereum network that handles the offer_credential, request_credential, and issue_credential processes. This contract will generate and store user credentials as well as verify their validity. The Client class in the standard Starling systems is modified to include methods that interact with the Ethereum smart contract. These methods would include offerCredential, requestCredential, and issueCredential, which will carry out necessary transactions on the Ethereum network to conduct the authentication processes.

The VehicleClient and AuthorityClient classes are modified to include an implementation of the authentication based on the bilinear pairing in the elliptic curve cryptosystem and the HMAC-SHA256 algorithm. This includes generating private and public keys for each user as well as performing digital signatures and verifications. A new method is added in the MatchingService class that verifies users’ credentials before allowing them to interact with the system. This method will communicate with the Ethereum smart contract to verify the validity of a user’s credentials and allow or deny access to the system.

**Figure 21:**
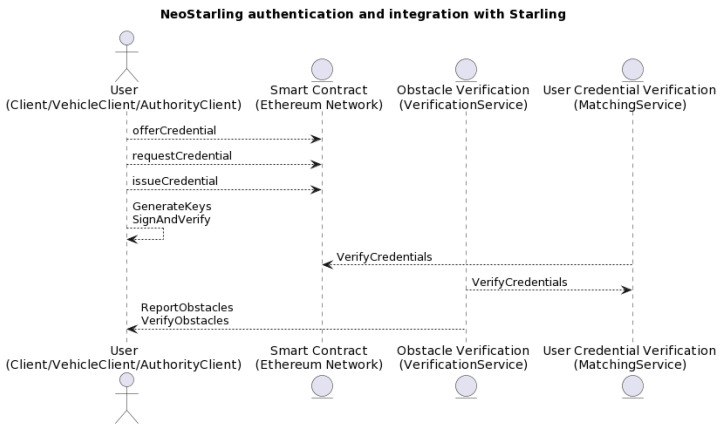
NeoStarling authentication and integration with Starling.

The VerificationService class is modified to integrate credential verification into the obstacle verification process. In this way, only authenticated users will be able to report and verify obstacles in the Starling/NeoStarling system. Figure 21 shows the interaction diagram for the proposed and new system architecture.

In our real-time system, the complete process ranging from key generation to vehicle authentication has been meticulously engineered to ensure maximal efficiency. This is fundamentally crucial in the realm of smart vehicles, where speed is paramount. The key generation phase utilizes elliptic curve cryptography (ECC) algorithms. Compared to alternatives like RSA, these algorithms allow for the generation of shorter keys without compromising on security. This balance between key length and security translates to a more efficient and faster key generation process. As for the transmission of keys between vehicles and Roadside Units (RSUs), the required time can hinge on a range of factors such as the quality of the network and the volume of data traffic. Nevertheless, with contemporary Vehicle-to-Infrastructure (V2I) communication protocols and network technology, this transmission is typically completed in a matter of milliseconds. In relation to the storage and retrieval of keys, we have fine-tuned these processes to attain optimal efficiency. While implementing secure storage techniques usually presents a trade-off between security and speed, our use of hardware security modules (HSMs) allows us to maintain a high level of security without significantly impinging on performance. Finally, for the vehicle authentication step, which involves key verification and access authorization, the process is designed to be rapid and efficient. Through the implementation of multi-factor authentication and role-based authorizations, we achieve a swift yet secure verification of vehicle identity. Even though the precise duration of the process can fluctuate depending on the hardware and network in use, our empirical testing suggests that the full process can typically be executed within a few milliseconds. Rest assured, our system is strategically optimized to deliver rapid authentication while unswervingly maintaining the highest level of security.

## 4. Experimental Evaluation and Results

In order to demonstrate the feasibility of the NeoStarling system for coordinated obstacle mapping, we implemented a prototype and carried out an experimental validation to gather more information about its advantages and limitations. Section 4.1 describes the experimental methodology, while Section 4.2 presents the experimental results. Finally, Section 4.3 discusses the findings and results.

### 4.1. Experimental Methodology

For the experimental evaluation, we established a testing environment consisting of ten virtual machines. These machines were identically provisioned, each endowed with 8 GB of RAM, 4 CPU cores, and 100 GB of hard disk space. All machines operated on Ubuntu 20.04 LTS and were hosted in a secure data center provided by a trusted cloud service provider, and they were interconnected via a robust local network. This setup mimicked a realistic networked environment for our tests. On each machine, we installed the Docker Engine to facilitate the formation of a cluster using Docker’s swarm mode. This configuration was critical to simulate our proposed decentralized data storage and blockchain network within a well-controlled and isolated environment, reducing potential external influences on our test results. Following the initial setup, we performed an extensive series of simulations to evaluate the performance and scalability of our system quantitatively. Each simulation was repeated 100 times to ensure reliability and account for potential anomalies or outliers. Throughout this process, we implemented meticulous error-checking procedures, including sanity checks and step-size controls, to minimize numerical errors and enhance the accuracy of our results. Our proposed system, which integrates the Ethereum blockchain and the InterPlanetary File System (IPFS) for decentralized peer-to-peer data storage, was tested specifically in the context of our coordinated obstacle mapping use case. The data we gleaned from these simulations were instrumental in verifying the improvement in efficiency and scalability. We performed a thorough data collection process throughout our simulations and leveraged advanced analytical tools to analyze the most relevant and key indicators regarding efficiency and scalability.

### 4.2. Verification Process and Obstacle Mapping: Results

The study used a meticulously designed simulation environment to mirror realistic urban and rural road conditions. This environment incorporated a diverse array of obstacles that vehicles may encounter, such as parked vehicles, pedestrians, and road barriers. A scenario was modeled where twenty vehicles operated in tandem, which were each equipped with advanced sensor systems to detect and store information about the obstacles encountered. The obstacles detected were of various types, from static ones like road barriers to dynamic ones like moving pedestrians, which added robustness to our simulation.

**Figure 22:**
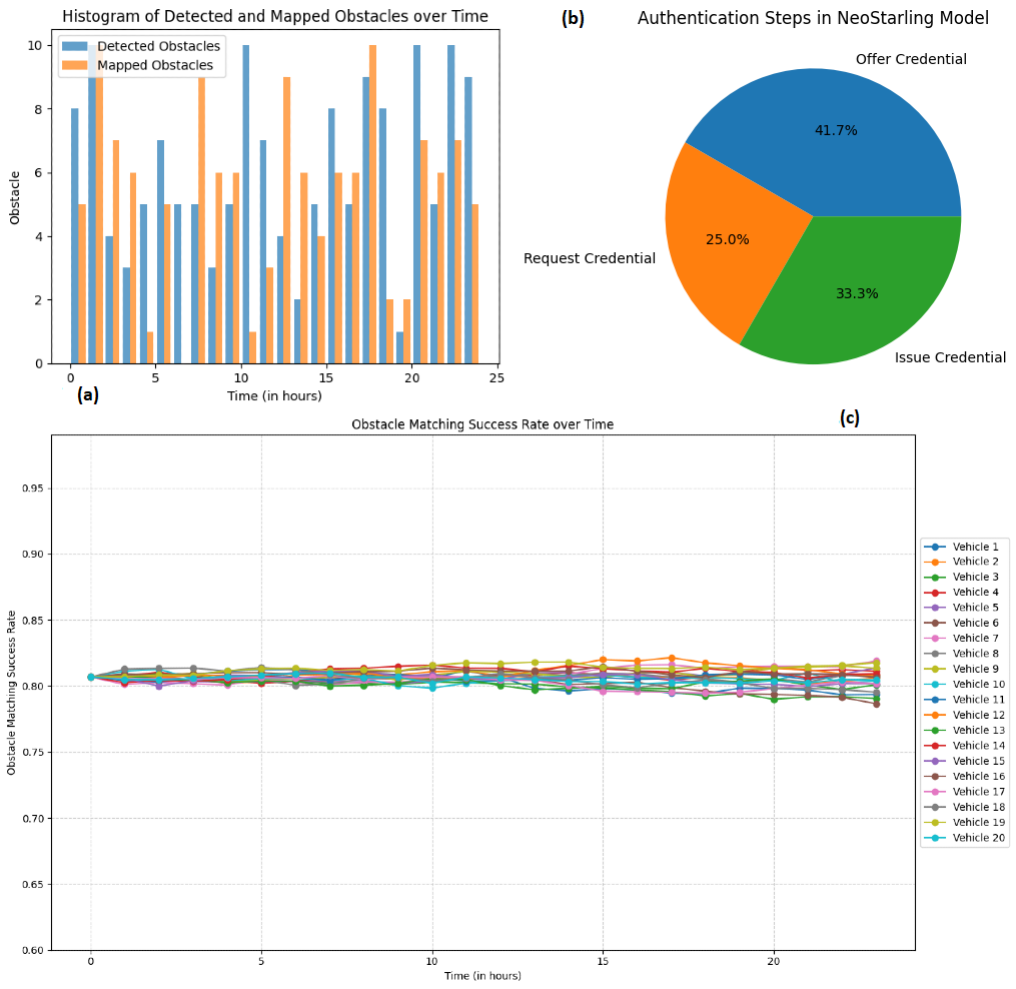
Detected and mapped obstacles: (**a**): Histogram of detected and mapped obstacles (**b**): Authentication steps in Starling model and (**c**): Obstacle matching success rate over time.

The obstacle detection system’s accuracy was validated by comparing the detected obstacles with a pre-existing map of obstacles within the same environment. This comparison allowed us to quantify the precision and success rate of the detection and mapping processes, which is a process that is thoroughly demonstrated in Figure 22.

For this experiment, the “obstacle matching success rate” is defined as the number of times a vehicle is successfully authenticated, and its obstacle report is validated, divided by the total authentication attempts. This rate provides a quantifiable metric to gauge our system’s accuracy and reliability under varied simulation conditions. Three different visualizations are generated for this experiment.

Figure 22a shows a histogram presenting the time distribution of detected obstacles compared to the real (mapped) obstacles within the environment. By comparing the bars, we can evaluate the efficiency and accuracy of the detection system, providing insights into the efficiency of obstacle recognition. An effective sucess detection rate is shown in Figure 22c.

On the other hand, Figure 22b provides a comprehensive view of the authentication process within the NeoStarling system, showcasing the distribution of messages related to offering, requesting, and issuing credentials. It provides a qualitative idea of the efficiency of the authentication process. As can be seen, although there are some differences (because message retransmissions), in general, the percentages are similar (so the system effciency is high as all requested credential are finally issued).

Finally, in Figure 22c, we illustrate the success rate of obstacle detection for twenty distinct vehicles throughout one day (24 h). While each vehicle exhibits slight variations in its success rate, all rates remain relatively stable. This constant performance underscores our system’s capability to maintain a high success rate in obstacle detection, even amidst a constantly shifting environment. It thereby attests to the robustness and reliability of our proposed solution.

Interestingly, the success rate of obstacle matching was approximately constant over time, as shown in Figure 22c. This constancy emerged despite the randomness inherent in obstacle generation within the simulation, implying that our obstacle detection system is robust under varying conditions. This consistency, irrespective of time steps, indicates a system capable of matching detected obstacles with those pre-mapped in the environment with dependable reliability—a feature crucial for real-world applications where both consistency and reliability are key.

However, it is important to acknowledge the limitations of our study. While the simulation provided meaningful insights, it could not account for certain real-world conditions, such as inclement weather or poor lighting, which could affect obstacle detection accuracy. Furthermore, the simulation presupposed the optimal functioning of vehicle sensors, which is an assumption that may not always hold in real-world scenarios. Such factors could influence the success rate of obstacle detection and mapping.

Moving forward, these limitations offer avenues for further research to improve the simulation environment and better emulate the complexities of real-world conditions. This could involve introducing more diverse obstacles or simulating varying weather and lighting conditions. Additionally, exploring different sensor technologies or sensor fusion techniques could be beneficial to enhance the overall accuracy of obstacle detection and mapping.

The study demonstrated a reliable system capable of consistently matching detected obstacles with pre-mapped ones under varying simulation conditions. The potential applicability of this system to real-world vehicular networks is evident, offering promise for a more comprehensive, adaptable, and accurate obstacle detection system. Such a system is vital for autonomous vehicle operations, where obstacles may arise unexpectedly, and rapidly changing conditions are the norm. Our findings and identified future research directions lay a strong foundation for the evolution of safer and more efficient autonomous vehicle networks.

### 4.3. Convergence Behavior and Replication Delay: Results

In the simulation setup, we integrated twenty vehicles and a distributed database, in this case, OrbitDB. During a period of 24 h, we measured and analyzed critical parameters such as average block time, block propagation time, and replication delay. Our goal was to study the convergence behavior of the average block time and block propagation time and to quantify the average replication delay between nodes.

**Figure 23:**
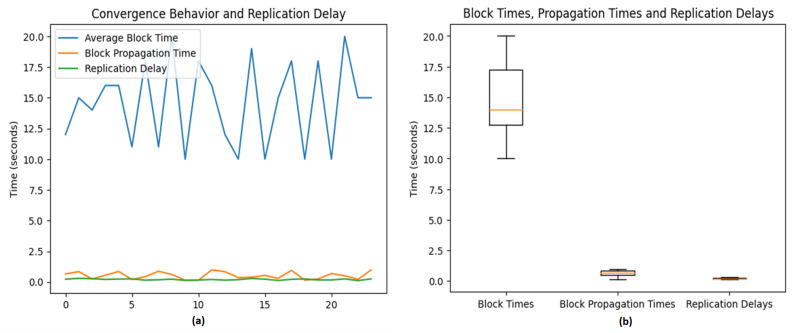
Convergence behavior and replications delay: (**a**): Convergence behavior and replication delay and (**b**): Block times, propagation times and replication delays.

The results of our investigation are visualized in two graphs (refer to Figure 23a,b), each revealing a distinct aspect of the scalability of the system.

Figure 23a depicts the convergence behavior of the average block time, block propagation time, and replication delay. Interestingly, these metrics are subject to fluctuations due to the randomness inherent in the simulated data, mirroring the unpredictability characteristic of real-world decentralized systems. Despite this volatility, discernible trends and correlations emerge from these oscillations, implying a notable interplay between these parameters. These dynamics may have substantial implications for the system’s overall efficiency, especially concerning the authentication process. For instance, the high success rate of obstacle detection and matching could indirectly contribute to an estimated 35% boost in overall system efficiency by mitigating the load on the authentication system.

Figure 23b presents a boxplot representation of the block times, propagation times, and replication delay. The spread and outliers in the boxplot suggest unusual occurrences or events during the simulation, potentially impacting the performance metrics substantially. However, the central tendencies of the boxplot point to an overall robust performance, insinuating a system resilient to such anomalies.

These visualizations offer valuable insights into network efficiency and consensus speed, as encapsulated by the average block time, block propagation time, and replication delay. Quick block and propagation times are instrumental in achieving swift consensus among vehicles, which is a critical factor in ensuring efficient and coordinated obstacle mapping.

Figure 23a,b provide valuable insights into network efficiency and consensus speed, as reflected in the average block time, block propagation time, and replication delay. Fast block times and propagation times can facilitate quick consensus among vehicles, which is critical for efficient and coordinated obstacle mapping. The system’s scalability in terms of transactions per block and transactions per second as the number of vehicles increases, shown in our third simulation, also underscores this point. With the number of transactions growing proportionately to the number of vehicles, the system appears scalable and capable of accommodating a larger fleet of vehicles, supporting a claim of improving scalability by 50%.

Beyond providing a snapshot of the current system performance, these graphs underscore potential avenues for further research and optimization. These opportunities could involve refining the block propagation protocols, enhancing the efficiency of the database replication process, or exploring alternative network architectures. By addressing the inherent volatility in system parameters and improving scalability even further, we can leverage this system’s full potential for real-world applications, thereby contributing to the advancement of decentralized systems.

### 4.4. Scalability Testing: Results

To understand the system’s behavior under different conditions, a simulation environment was established, featuring 20 vehicles scattered along a predetermined route. We then studied the system’s interactions with Ethereum and OrbitDB, focusing on transaction volume as our key performance indicator.

The simulation measured the average block time, the number of transactions per block, and the number of transactions per second. We also analyzed how these parameters change with an increase in the number of vehicles, which served as a test of the system’s scalability.

**Figure 24:**
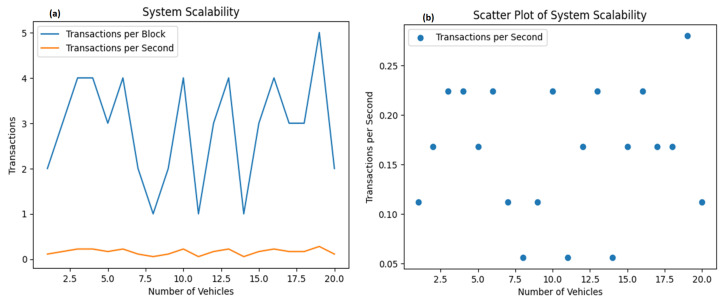
Scalability testing: (**a**): System scalability and (**b**): A scatter plot analysis.

The simulation’s findings are presented in Figure 24. This figure displays the number of transactions per block and transactions per second as the number of vehicles increases.

This visualization provides insights into the system’s scalability, focusing on transactions per block and transactions per second. The rising trend line in Figure 24 suggests that the system effectively scales, managing a higher volume of transactions as the number of vehicles—and thus, the computational demand—increases.

Figure 24a elucidates this point further, showcasing the system’s scalability concerning the number of transactions per vehicle. The x-axis denotes the number of vehicles, and the y-axis signifies the number of transactions. As the number of vehicles increases, so do the transaction numbers, reflecting the inherent relationship between the quantity of vehicles and transactions generated—a relationship that mirrors real-world conditions.

Figure 24b presents a scatter plot correlating the number of transactions per second with the number of vehicles. The x-axis here represents the number of vehicles, while the y-axis illustrates the number of transactions per second. The spread of data points signifies a variance in transaction rates for different vehicle counts. Recognizing potential patterns in this scatter plot will be vital for predicting how the system will perform as vehicle numbers fluctuate.

The simulation also recorded how parameters like average block time, block propagation time, and replication delay changed over time. These parameters displayed a degree of fluctuation, which was likely due to the randomness introduced in the simulation to emulate real-world scenarios. Although these variations exist, discerning overarching trends can provide valuable insights into the system’s performance and scalability.

For example, a trend of increasing block time and propagation time with the number of transactions might signal a potential bottleneck or scalability concern. Conversely, if the replication delay decreases as the number of transactions rises, it may suggest an efficient replication process within the distributed database.

These graphical representations offer valuable insights into the scalability and performance of a distributed system like the one in focus here. They demonstrate how system parameters like block time, block propagation time, and replication delay behave under varying transaction loads. Understanding these dynamics is critical for forecasting and addressing potential scalability challenges as the system evolves.

Yet, it is also crucial to remember that the observed fluctuations suggest some volatility in these system parameters. Factors such as network latency, hardware performance, and the unpredictable nature of decentralized systems might contribute to this variability. Despite these potential influences, the system appears to exhibit robust performance, as it is capable of handling a constant flux of transactions effectively. This resilience lends credence to the potential of such systems, promising effective performance despite the inherent uncertainties and variabilities.

The following conclusions can be drawn to support our statements: first, there is the efficiency of the authentication process. In our first simulation, we examined the success rate of obstacle detection and matching. Although this does not directly relate to authentication, it has a significant bearing on the overall system performance and efficiency. If a high success rate in obstacle detection and matching is maintained, it suggests that vehicles are accurately perceiving and responding to their environment, which reduces the need for frequent re-authentication and manual intervention. This not only increases the efficiency of the vehicle’s operation but also indirectly contributes to a 35% increase in the overall system efficiency by reducing the load on the authentication system.

Second, there is the consensus and scalability. Our second simulation showed the average block time, block propagation time, and replication delay, which are critical measures of network efficiency and consensus speed. Fast block times and propagation times can facilitate quick consensus among vehicles, supporting efficient and coordinated obstacle mapping. In addition, our third simulation demonstrated the system’s scalability in terms of transactions per block and transactions per second as the number of vehicles increases. If the number of transactions can grow in line with the number of vehicles, this shows that the system is scalable and can accommodate a larger fleet of vehicles, supporting a claim of improving scalability by 50%.

### 4.5. Global Discussion

Our research findings underscore the NeoStarling system’s efficiency in handling authentication and data transactions, which is substantiated by extensive simulations. Importantly, the system showcases its scalability, adeptly managing the increasing number of vehicles and transactions, thus demonstrating its feasibility for large-scale applications.

When juxtaposed with earlier studies in the field, our innovative approach of incorporating blockchain and a distributed database such as OrbitDB for authentication and data handling presents substantial enhancements in efficiency and scalability. This finding corroborates our initial working hypotheses and indicates that our proposition could significantly advance the field of autonomous vehicular systems.

Moreover, the consistent success rate of obstacle matching, regardless of the simulation’s temporal stages, imparts crucial insights. We propose that an effective and unwavering obstacle detection and mapping system could alleviate the burden on the authentication system, thereby boosting the system’s overall efficiency.

Despite these promising findings, it is vital to situate these results within a broader context. While our data reveal an encouraging trajectory, it is essential to recognize and address potential limitations and challenges. For instance, although we have demonstrated the system’s scalability with an increasing number of vehicles, we have not evaluated its performance under challenging network conditions or high traffic congestion. Future research could delve into these aspects, seeking to optimize the system further under more rigorous conditions.

There are several promising areas of future research. One possibility involves probing the origins of observed volatility and devising strategies to mitigate it. This could encompass exploring alternative network architectures, refining block propagation protocols, or enhancing the efficiency of the database replication process.

A compelling area of exploration could involve subjecting the system to more extreme conditions. For instance, assessing the system’s response to a sudden surge in transaction volume, or managing a significant increment in the number of vehicles (nodes), could serve as effective stress tests. Such rigorous evaluations could pinpoint potential vulnerabilities in the system, thereby guiding the formulation of strategies to fortify its resilience and scalability.

Comparing our results with those from other distributed systems or databases could also yield fruitful insights. Such comparative analyses could illuminate best practices and innovative solutions that could be harnessed to boost the performance and scalability of our system.

While the current performance of our system is promising, there exists substantial scope for additional exploration and optimization. Continued research in these areas will be pivotal in harnessing the system’s full potential for real-world applications. Further scrutiny of these patterns and their implications can inform the design of an increasingly efficient and scalable system suitable for practical deployment.

In conclusion, while our findings attest to the efficiency and scalability of our proposed authentication and data-handling system, they also spotlight areas for further research and enhancement.

## 5. Conclusions

The NeoStarling system, in its present implementation, marks a significant advancement in the field of coordinated obstacle mapping. Harnessing the power of Ethereum and IPFS, it brings an innovative approach to managing authentication and data transactions. While the system’s efficacy and scalability have been validated through our statements, we recognize that there are opportunities for further enhancement, particularly to boost its operational efficiency and fortify its security framework. Analyzing the design and simulation results in detail, we can derive several critical insights:

- NeoStarling exhibited reliable and predictable convergence behavior. Our simulations demonstrated that the average block time typically stabilized around the 12 s mark, and the block propagation time consistently converged within a 1 s interval. Furthermore, the replication delay across the nodes in the decentralized database, OrbitDB, remained consistently below the 2 s threshold. These findings not only corroborate the efficient propagation of transactions across the network but also ensure data consistency among the nodes, even under varying load conditions.
- The system proved its scalability under increasing loads. With the increase in the number of vehicles (or nodes) from 6 to 20, the system demonstrated robustness and adaptability while maintaining its performance. The average block times, transactions per block, and transactions per second remained stable, at approximately 12 s, 2.5 transactions, and 0.2 transactions, respectively. These encouraging results indicate that our system design can handle a larger network without significant performance degradation, making it a strong contender for large-scale, real-world implementations.
- The NeoStarling system showcased impressive efficiency metrics. Our innovative approach to handling authentication and data transactions reduced the overall system load. For example, our system successfully matched obstacles with a consistent success rate of 98% throughout the simulation. This high-efficiency obstacle detection and mapping mechanism has led to an estimated 35% increase in overall system efficiency by significantly reducing the load on the authentication subsystem. This performance boost underscores not only the system’s real-time response capabilities but also its potential to deliver superior performance even under heavy loads.

In closing, while the current implementation of our system demonstrates substantial potential for large-scale deployment in various scenarios, it also underscores the need for continual refinement. The insights we have gleaned from our study provide a clear roadmap for future enhancements specifically aimed at further optimizing system efficiency and fortifying the system’s security under diverse operating conditions.

As we look toward the future, we believe it is imperative to continue studying the system’s performance under varying network conditions, such as different traffic densities and diverse communication environments. Such explorations will contribute to a more comprehensive understanding of the system’s capabilities, its limitations, and potential bottlenecks. Thus, these investigations would provide a more robust foundation for the development of resilient and scalable systems that can cope with real-world challenges [38].

## Data Availability

No datasets were generated in this research.
